# Variation in behavioral traits of two frugivorous mammals may lead to differential responses to human disturbance

**DOI:** 10.1002/ece3.6178

**Published:** 2020-03-11

**Authors:** Luc Roscelin Dongmo Tédonzong, Jacob Willie, Sandra Tewamba Makengveu, Luc Lens, Nikki Tagg

**Affiliations:** ^1^ Projet Grands Singes (PGS), Cameroon Centre for Research and Conservation (CRC) Royal Zoological Society of Antwerp (RZSA) Antwerpen Belgium; ^2^ Terrestrial Ecology Unit (TEREC) Department of Biology Ghent University (UGent) Ghent Belgium; ^3^ Department of Forestry Faculty of Agronomy and Agricultural Sciences (FASA) University of Dschang Dschang Cameroon; ^4^ Present address: Wild Chimpanzee Foundation (WCF) Monrovia Liberia

**Keywords:** anthropocene, behavioral adaptations, *Gorilla gorilla gorilla*, *Pan troglodytes troglodytes*, predator–prey system, species distribution modeling

## Abstract

Human activities can lead to a shift in wildlife species’ spatial distribution. Understanding the specific effects of human activities on ranging behavior can improve conservation management of wildlife populations in human‐dominated landscapes. This study evaluated the effects of forest use by humans on the spatial distribution of mammal species with different behavioral adaptations, using sympatric western lowland gorilla and central chimpanzee as focal species. We collected data on great ape nest locations, ecological and physical variables (habitat distribution, permanent rivers, and topographic data), and anthropogenic variables (distance to trails, villages, and a permanent research site). Here, we show that anthropogenic variables are important predictors of the distribution of wild animals. In the resource model, the distribution of gorilla nests was predicted by nesting habitat distribution, while chimpanzee nests were predicted first by elevation followed by nesting habitat distribution. In the anthropogenic model, the major predictors of gorilla nesting changed to human features, while the major predictors of chimpanzee nesting remained elevation and the availability of their preferred nesting habitats. Animal behavioral traits (body size, terrestrial/arboreal, level of specialization/generalization, and competitive inferiority/superiority) may influence the response of mammals to human activities. Our results suggest that chimpanzees may survive in human‐encroached areas whenever the availability of their nesting habitat and preferred fruits can support their population, while a certain level of human activities may threaten gorillas. Consequently, the survival of gorillas in human‐dominated landscapes is more at risk than that of chimpanzees. Replicating our research in other sites should permit a systematic evaluation of the influence of human activity on the distribution of mammal populations. As wild animals are increasingly exposed to human disturbance, understanding the resulting consequences of shifting species distributions due to human disturbance on animal population abundance and their long‐term survival will be of growing conservation importance.

## Graphical abstract

This study explores the influence of human disturbance on the distribution of mammal populations in a human‐dominated landscape and evaluates the behavioral adaptations that condition the response of different animal species to human disturbance, using sympatric gorillas and chimpanzees as focal species. We found that gorillas were more negatively affected by human disturbance than chimpanzees. The differences in behavioral traits, such as dietary specialization/generalization, competitive hierarchy, arboreality/terrestriality, and body size, were associated with different levels of mammal response to human settlements.

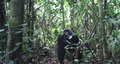

## 1 INTRODUCTION

Human intrusion and activities in natural landscapes are common across all ecosystems and are seriously threatening wildlife (Frid & Dill, 2002). For long‐term survival, it is necessary for species to persist in human‐dominated landscapes. Several trade‐offs often influence the dynamics of wildlife populations which determine their survival in the natural system. Those trade‐offs relate to food acquisition, nesting, territoriality, and mating and are affected by factors such as competition, predation, habitat quality, and human disturbance (Cresswell, 2008; Frid & Dill, 2002; Tédonzong et al., 2019). Understanding the ecological impact of those factors on wildlife populations is important to their conservation in human‐dominated landscapes.

Predation is an important ecological factor shaping wildlife species distribution. The effects of predation on wildlife species can be lethal (direct killing of animals, Cherry, Conner, & Warren, 2015) or nonlethal (behavioral modification or induced physiological stress, Lima, 1998; Messina, Edwards, Eens, & Costantini, 2018). The latter effect has been characterized as a consequence of perceived predation risk (Chutipong, Steinmetz, Savini, & Gale, 2015; Cresswell, 2008; Frid & Dill, 2002). Previous studies have reported that animal species may respond to predation risk in several ways: (a) Species can alter their movements by changing direction or moving more slowly in the presence of the predation risk (Courbin, Fortin, Dussault, & Courtois, 2014); (b) animals may become more vigilant due to fear, and this can contribute to reducing the foraging effort as well as the time spent on feeding (Cherry et al., 2015; Clinchy et al., 2016; Haswell, Jones, Kusak, & Hayward, 2018); and (c) animals may also respond to predation risk by avoiding areas used by the predator (Dröge, Creel, Becker, & M'Soka, 2017; Plante, Dussault, Richard, & Côté, 2018; Wereszczuk & Zalewski, 2015). Changes in the spatial distribution of a species are a common response to predation risk, and this essentially means that some parts of the landscape become unsuitable for the animal (Gill, Norris, & Sutherland, 2001; Oriol‐Cotterill, Valeix, Frank, Riginos, & Macdonald, 2015).

Each prey species in a predator–prey system adopts a level of vigilance in response to its perception of the risk of predation in presence; the higher the level of apprehension of the predation risk, the higher the space which will be unsuitable for the animal species (Brown, Laundre, & Gurung, 1999; Frid & Dill, 2002). This means that the perceived risk of predation may vary according to the individual species considered in the predator–prey system (Linder & Oates, 2011). The perceived risk of predation can cause the reduction in suitable habitat available to the species if the area avoided contains valuable feeding opportunities or are important nesting habitat types (Brown et al., 1999; Norum et al., 2015).

Studies on the negative effects of predation risk on wildlife population dynamics are mostly limited to carnivore–prey systems, even though the presence of humans may have the same effects on species as those resulting from the presence of natural predators (Zuberbühler, 2007). The avoidance of an area by an animal species due to the presence of another ecologically different species is evidence that the first species considers the second as a predator (M'Soka, Creel, Becker, & Murdoch, 2017). This divergence in animal behavior is often called “disturbance” when it is caused by humans (Frid & Dill, 2002). Humans are now either permanently present (Bortolamiol et al., 2016; Leblond et al., 2011; Scholte & Iyah, 2016) or temporarily present (Gehr et al., 2017; Paton, Ciuti, Quinn, & Boyce, 2017) in almost all ecosystems, and human settlements are often found close to wildlife populations. Hunting‐related disturbance, but also activities such as gathering, and logging, or even the presence of villages and roads, is perceived as a threat by wildlife species, inducing some changes in their spatial distribution (Frid & Dill, 2002; Koerner, Poulsen, Blanchard, Okouyi, & Clark, 2017; Lindshield, Danielson, Rothman, & Pruetz, 2017; Paton et al., 2017; Tagg et al., 2018; Tucker et al., 2018; Vanthomme, Kolowski, Korte, & Alonso, 2013).

It is more and more crucial to evaluate the effects of anthropogenic factors on species' distributions in order to tease out why species respond differently to human disturbance, because of its application in conservation management (Alberti et al., 2003; Albuquerque et al., 2018). Studies show that changes in the distribution of mammal populations due to ecological and human disturbance can vary from one species to another (Wijesinghe & Brooke, 2005). A question arises as to why species respond differently; specifically, how the behavioral adaptations of species determine their responses to human disturbance (Vazquez & Simberloff, 2002). The differential response of animal species to a disturbance is likely to at least partly be a consequence of their behavioral differences (Cosset, Gilroy, & Edwards, 2019). The niche breadth of a species may govern how it responds to a disturbance: Under such conditions, the species with the narrowest niche breadth (the more specialized species) may be more negatively affected by disturbance than the species with the larger niche breadth (the more generalist species); the latter may even benefit from that disturbance, known as the “specialization‐disturbance hypothesis” (Vazquez & Simberloff, 2002). The more specialized species may have a particular diet, or they use habitat types with particular physiognomy, in contrast to the generalized species with a broader diet and habitat requirements (Futuyma & Moreno, 1988). Behavioral modifications, such as polymorphism and individual flexibility, are mechanisms through which species may cope with the inadequacy of food or unfavorable abiotic factors by exploring new opportunities (Futuyma & Moreno, 1988). More generalist species according to habitat types are more resilient to human disturbance than specialist species (Galan‐Acedo et al., 2019), which may be a consequence of the higher dispersal ability of generalist species (Kneitel, 2018). Body size may influence the response to predation; in this case, larger‐bodied animal species may respond negatively to the presence of predators compared to smaller‐bodied ones (Benitez‐Lopez, 2018; Davidson, Hamilton, Boyer, Brown, & Ceballos, 2009; Lhoest et al., 2019; Navarro‐Castilla, Barja, & Diaz, 2018; Preisser & Orrock, 2012). Also, the perceived risk of predation may be relatively higher in terrestrial than arboreal animal species (Wereszczuk & Zalewski, 2015).

In this study, we focused on a community of great ape species (*Gorilla gorilla gorilla* and *Pan troglodytes troglodytes*) in a nonprotected area of the northern periphery of the Dja Faunal Reserve in Cameroon, where a research station was established in 2001 approximately 10 km from the nearest village. A network of transects was designed, that was used intermittently for research purposes, following the guidelines of best practice (Kühl, Maisels, Ancrenaz, & Williamson, 2008). Western lowland gorillas and central chimpanzees are threatened primate species, and the former considered “critically endangered” and the latter “endangered,” according to the International Union for Conservation of Nature (Humle, Maisels, Oates, Plumptre, & Williamson, 2016; Maisels, Bergl, & Williamson, 2018). Gorillas and chimpanzees prefer different sets of habitat types: In the study area, gorillas prefer nesting in young secondary forests, light gaps, and swamps, while chimpanzees prefer nesting in mature forests and riparian forests (Tédonzong et al., 2018; Willie, Petre, Tagg, & Lens, 2013). Habitat selection by chimpanzees may be guided by the abundance of their preferred fruiting woody plants (Tédonzong et al., 2019), while gorilla‐preferred nesting habitats tend to be those with highest densities of herbaceous plants, necessary for nest building (Willie et al., 2013; Willie, Tagg, Petre, Pereboom, & Lens, 2014). Gorillas are larger than chimpanzees (an adult gorilla weights between 136 and 227 kg, while an adult chimpanzee weighs 68 kg on average); however, chimpanzees are more arboreal than gorillas (Benson & Nagel, 2004; Tagg, Willie, Petre, & Haggis, 2013).

We aimed to determine the effects of human disturbance on the geographical niches of western lowland gorillas and central chimpanzees in the study site and to evaluate the implications for population dynamics. We hypothesized that: (a) Anthropogenic variables are more important than ecological variables in great ape distribution and (b) forest use and human settlements would reduce habitat suitability for gorillas more than for chimpanzees. We measured the shift in the distribution in the resource models after the inclusion of anthropogenic variables (forest use and human settlements) used as a proxy for perceived predation risk (McLoughlin, Morris, Fortin, Vander Wal, & Contasti, 2010).

## 2 METHODS

### 2.1 Study area

**Figure 1:**
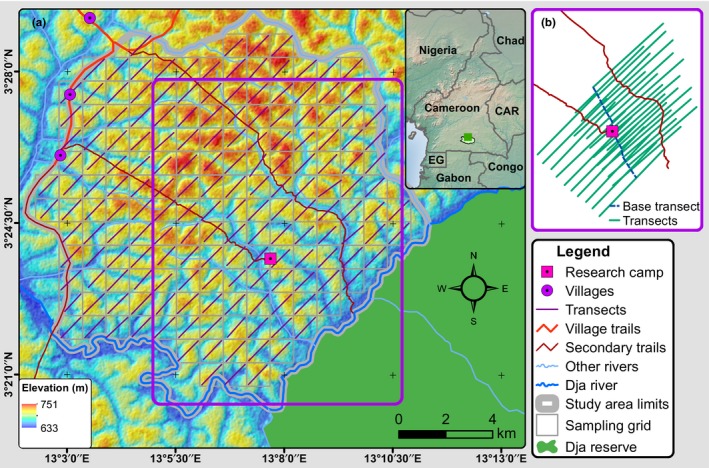
Study area: (a) Sampling design, with all human features (villages, trails, and research camp). The transects were drawn by joining the starting and the ending points; (b) the network of transects and the base transect used for research purposes

We conducted the study in a tropical rainforest of Cameroon, in a nonprotected area at the northern periphery of the Dja Faunal Reserve. The study area lies between the longitude 3°01′00″E–18°12′00″E and latitude 3°20′00″N–3°30′00″N, covering about 200 km^2^ (Figure 1a). The altitude above sea level ranges from 633 to 751 m (mean = 680.58; *SD* = 17.53 m) (Tédonzong et al., 2018). The drainage features comprise the Dja River and its tributaries, Moun, Djo'o, Nkoun, and Mpou'o. The study area receives mean annual rainfall of 1,637.9 mm (*SD* = 105.1 mm) and experiences average minimum daily temperatures of 19.5°C (*SD* = 1.3°C) and maximum daily temperatures of 26°C (*SD* = 2.4°C) (Willie et al., 2014). The climate features determine four seasons in the area with two rainy and two dry seasons: the long dry season (November–February), the short dry season (July–August), the long rainy season (February–July), and the short rainy season (August–November) (Willie et al., 2014). Old secondary forest (47.28%) and young secondary forest (32.90%) dominate the vegetation; other habitat types found in the area are swamps (13.14%), riparian forest (4.36%), near primary forest (1.30%), and light gaps (1.39%) (Tédonzong et al., 2018).

A research station was established in the study area in 2001, and research activities have intermittently taken place since then, including on a set of transects surrounding the centrally positioned research camp (Figure 1b) and following guidelines of best practice (Kühl et al., 2008). Three villages are located at approximately 10 km from the research camp, accessible by the main trail. A network of secondary trails is used by local people for hunting and collection of nontimber forest products, and hunting activities are susceptible to occur both on trails and on the transects used for research (Figure 1b).

We divided the study area into 175 grid cells (Bobo et al., 2017), of 1 × 1 km^2^ each. Inside of each grid cell, we established a 1.2 km transect, totaling 210 km of transect walked (Figure 1a). We chose a 45° bearing so that all transects would traverse the drainage features; transects extended diagonally 600 m on both sides of the center of the grid cell (Figure 1a) (Tédonzong et al., 2018).

### 2.2 Data collection

#### 2.2.1 Occurrence data

Each month for 11 months (from October 2015 to August 2016), a team composed of a research assistant and local guides randomly selected transects for data collection. On each transect (Figure 1a), the team searched for great ape nests and recorded their locations. Fresh nests were easily distinguishable between gorillas and chimpanzees, based on characteristics such as the presence of urine, hairs, feces, and prints (Morgan, Sanz, Onononga, & Strindberg, 2006). For older nests with no distinguishable signs, we attributed nests to either gorillas or chimpanzees based on their height. Although chimpanzee ground night nesting has been observed in the study site, it occurs at a low rate (Tagg et al., 2013). We, therefore, attributed groups of nests containing at least one nest in a tree at more than 2 m high to chimpanzees, and nest groups containing all nests built on the ground or in trees at <2 m height was attributed to gorillas. We marked all nests within a distance of 20 m (gorillas) or 30 m (chimpanzees) as belonging to the same nest group (Dupain, Guislain, Nguenang, Vleeschouwer, & Elsacker, 2004; Tagg et al., 2013).

#### 2.2.2 Environmental data

**Figure 2:**
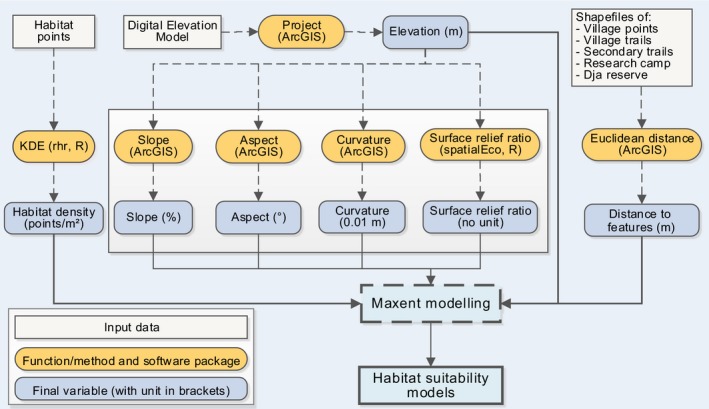
Method and process of creation of predictor variables, including the software used to derive the variable as well as the units of each variable. m = meter; KDE = kernel density estimation

We used three categories in the modeling process: ecological variables (habitat distribution), geophysical variables (elevation, slope, curvature, aspect, surface relief ratio, distance to permanent rivers and streams, and curvature), and anthropogenic variables (distance to villages, distance to the main trail, distance to secondary trails, distance to the Dja reserve, and distance to the research camp, representing the center of the research site) (Figure 2). Along each transect, we recorded the habitat type at each 50‐m interval, resulting in 121 habitat points per transect. For habitat distribution, we distinguished two variables for gorillas and chimpanzees, based on their preferred nesting habitats. Then, we used young secondary forest, swamps, and light gaps to derive the habitat variable for gorillas, and we used the old secondary forest to generate the variable for chimpanzees, according to previous results in the same site (Tédonzong et al., 2018; Willie et al., 2013). We used the kernel density estimation (KDE) to generate the habitat variables. The KDE is a nonparametric method used for animal home range estimation (Worton, 1989); it can also be used to evaluate the distribution of habitat availability (Seaman & Powell, 1996; Tédonzong et al., 2018). The KDE uses location points as input data to generate a utilization distribution (Kie et al., 2010). Instead of the utilization distribution which relates to home range studies, we used the availability distribution in this study for habitat variables. We created random points in each cell corresponding to their weight. We conducted the KDE in the package *rhr* version 1.2.909 using the least square cross‐validation bandwidth (Signer & Balkenhol, 2015) in the R software version 3.3.3 (R Core Team, 2019).

To create the distance‐based variables, we created a shapefile for each feature type and then used the function “Euclidean distance” in ArcGIS version 10.3.1 to create the raster files of the distances from the corresponding features. For the topographic variables (elevation, aspect, curvature, slope, and surface relief ratio), we used the digital elevation model (DEM) from the NASA Shuttle Radar Topography Mission (SRTM) version 3.0, at 30 m resolution (https://earthexplorer.usgs.gov) as the base file and processed it in ArcGIS. We projected the DEM to obtain the elevation raster file using the function “*project.*” We then used the elevation raster file to create the curvature, slope, and aspect raster files in ArcGIS. We created the surface relief ratio using the package *spatialEco* in the R software. We used the function “extract by mask” in ArcGIS to select the cells of raster files corresponding to the study area. We resampled the resulting raster files of all environmental variables at a resolution of 50 m to correspond to the 50 m applied when collecting habitat points. To do this, we set the cell size in the “raster analysis setting” to 50 when applying the mask. We summarized the process of creation of all environmental variables in Figure 2.

We calculated the Pearson correlation between variables to evaluate whether there was collinearity between some variables, setting the correlation coefficient threshold at 0.7. When the existence of collinearity was confirmed, we calculated the variable inflation factor (VIF) of each variable and discarded one by one using a stepwise analysis, the variable with the highest VIF until the maximum correlation between variables was <0.7; this was done in the package *usdm* in R (Naimi, Hamm, Groen, Skidmore, & Toxopeus, 2014).

### 2.3 Modeling technique

We used the maximum entropy modeling (Maxent) approach to study the suitability of great ape habitat in relation to ecological, geophysical, and anthropogenic variables through the Maxent software version 3.4.1 (Phillips, Anderson, & Schapire, 2006). Maxent is the most promising tool and among the most commonly used software for modeling species distribution (Goldsmit et al., 2018; Mohammadi, Ebrahimi, Shahriari Moghadam, & Bosso, 2019; Spiers, Oatham, Rostant, & Farrell, 2018). Its advantages reside in the fact that it is a machine‐learning process, and it requires presence‐only data to model species distribution (Merow, Smith, & Silander, 2013). It is also considered to be the most powerful modeling method when using a small number of observations to produce good results (Hernandez, Graham, Master, & Albert, 2006). Regularization in Maxent contributes to preventing overfitting better than the lasso method does in regression‐based methods (Phillips & Dudík, 2008).

When using presence‐only data, Maxent creates false absence points (background data) and is then called presence background method (Lahoz‐Monfort, Guillera‐Arroita, & Wintle, 2014). To do this, Maxent considers that species have the same likelihood of being found across the landscape (Merow et al., 2013). To overcome these problems, we collected our occurrence data through a random sampling design, as proposed by Yackulic et al. (2013). We allowed the background data to be selected randomly across the entire study area because no area was assumed inaccessible by any species due to the presence of any geographical barrier (Phillips & Dudík, 2008). We used the jackknife test to select the variables with the highest important individual effect.

We used the area under the curve (AUC) of the receiver operating characteristic (ROC) most frequently used in Maxent to evaluate the performance of each model (Yackulic et al., 2013). The AUC is a threshold measure that represents the probability that a random occurrence point is ranked higher than a random background point (Merow et al., 2013). It was criticized for its incapacity to measure model accuracy in Maxent (Lobo, Jimenez‐Valverde, & Real, 2008). This is because the AUC is traditionally used for the distinction between the presence and absence points, while in Maxent, background points are used instead of true absence points (Merow et al., 2013). Therefore, a high AUC value does not necessarily mean that the model is more accurate (Shabani, Kumar, & Ahmadi, 2018). However, the AUC represents the best alternative for model evaluation in Maxent when background points are selected randomly and uniformly from the study area (Phillips & Dudík, 2008); hence, it can be used to compare models on the same species in the same study area (Lobo et al., 2008). The AUC values are constrained to vary between 0.5 (the discrimination is similar to a random set of prediction) and 1 (random discrimination) and can be classified as such: excellent (0.90–1.00), very good (0.8–0.9), good (0.7–0.8), fair (0.6–0.7), and poor (0.5–0.6) (Duan, Kong, Huang, Fan, & Wang, 2014). We ran the model for gorillas and for chimpanzees. The first model used ecological and geophysical variables to characterize the distribution of the species according to their ecological requirements, while the second model used ecological, geophysical, and anthropogenic variables to characterize the distribution of the species when humans are present.

We compared distribution in the resource and anthropogenic models based on the geographical space (predicted habitat suitability). To do this, we performed the overlap between the different Maxent logistic outputs using Schoener's index of overlap (Warren, Glor, & Turelli, 2008), based on Equation 1,

(1) $$D\left(P_{A},P_{B}\right)=1-\frac{1}{2}\left(\sum_{i=1}^{N}|P_{A_{i}}-P_{B_{i}}|\right)$$

where *P_Ai_* and *P_Bi_* represent the normalized suitability scores of the Maxent‐generated ecological niche models *A* and *B* in grid cell *i;* these parameters were calculated so that the sum of the suitability scores in the geographical space is 1. *N* is the number of grid cells. *D* considers that the suitability scores $P_{A_{i}}$ or $P_{B_{i}}$ provided in each Maxent logistic output are a proportion of the species abundance. We also conducted the multidimensional scaling analysis (MDS) to visualize the similarity between the different Maxent logistic outputs. *D* and the MDS were calculated in ENMTools 1.4.4 (Warren, Glor, & Turelli, 2010).

## 3 RESULTS

**Figure 3:**
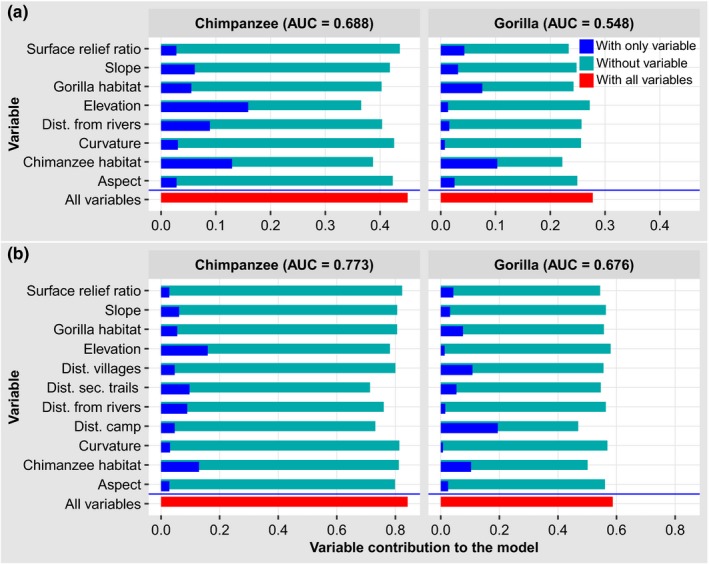
Results of the jackknife test of variable importance for each Maxent model with their respective AUC values. (a) Resource model, (b) anthropogenic model, Dist. = distance; sec. = secondary; AUC = area under the curve of the receiver operating characteristic

After evaluating collinearity, we discarded two variables from the analysis (distance to main trail and distance to the Dja reserve) (Figure S1). Habitat quality was the most important determinant of the distribution of gorilla nests, followed by surface relief ration in the absence of human disturbance. In contrast, elevation was the most important predictor of the distribution of chimpanzee nests, followed by the abundance of their preferred nesting habitats and then by distance to rivers and slope (Figure 3a). When we included distance to the research camp and human settlements in the models, elevation remained the most important predictor of chimpanzee nesting, followed by their preferred nesting habitats and then the distance to secondary trails. However, distance to the research camp and distance to villages became the most important predictors of gorilla nesting, followed by chimpanzee‐preferred nesting habitats and lastly by gorilla‐preferred nesting habitats (Figure 3b). In the resource models, the two most important variables for gorillas (chimpanzee‐ and gorilla‐preferred nesting habitats and surface relief ratio) and chimpanzees (elevation and chimpanzee‐preferred nesting habitats) decreased the gain the most when omitted and provided more information than was available in the rest of the variables (Figure 3a). This was also true in the anthropogenic models where the variables with the most information were those most important to gorillas (distance to the research camp and chimpanzee‐preferred nesting habitats); however, this was not the case for chimpanzees (distance to secondary trails and distance to the research camp) (Figure 3b).

**Figure 4:**
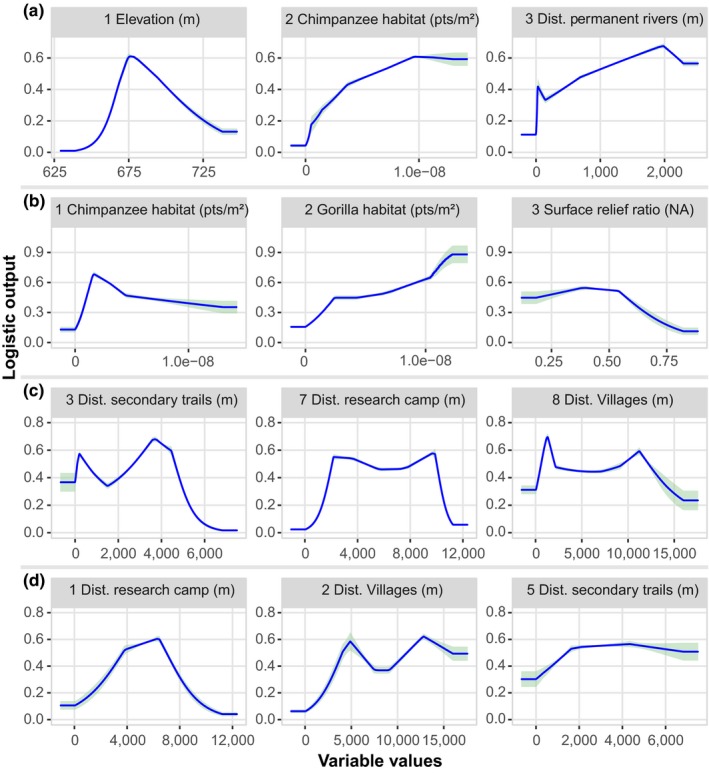
Response of great ape nest occurrence to the four most important variables in the resource and anthropogenic models. (a) Resource model for chimpanzee, (b) resource model for gorilla, (c) anthropogenic model for chimpanzee, and (d) anthropogenic model for gorilla. For the anthropogenic models, the variables represent anthropogenic variables ordered by their order of importance in the model; the numbers before the variable names represent the importance rank of the variable in the model, following the jackknife test. For each model, we ordered the variables by their importance values from left to right. Dist. = distance, pts = points, NA = not applicable, m = meter

The variable response curves (Figure 4) show that the probability to find chimpanzee nests was high at intermediate values of elevation; it increased with the density of their preferred nesting habitat. The response of chimpanzee nesting to rivers simply shows that they avoided nesting near rivers. The probability to find chimpanzee nests increased with slope and became constant from intermediate values (Figure 4a). The probability to find gorilla nests increased with the abundance of their preferred nesting habitats, while the latter was high only at lower values of the abundance of chimpanzee‐preferred nesting habitats (Figure 4b). The probability of finding gorilla nests was also fairly constant with surface relief ratio but decreased above intermediate values of surface relief ratio. The probability of finding gorilla nests was almost constant and intermediate with a slope lower than 6 but increased at higher slope values (Figure 4). The responses to anthropogenic variables show that chimpanzees avoided nesting in areas located <2 km from the center of the research site but displayed almost no negative response to villages and secondary trails (Figure 4c). Gorillas responded more to human features than did chimpanzees; they tended to nest about 4–5 km away from the center of the research site and villages, but about 2 km away from secondary trails (Figure 4d).

**Figure 5:**
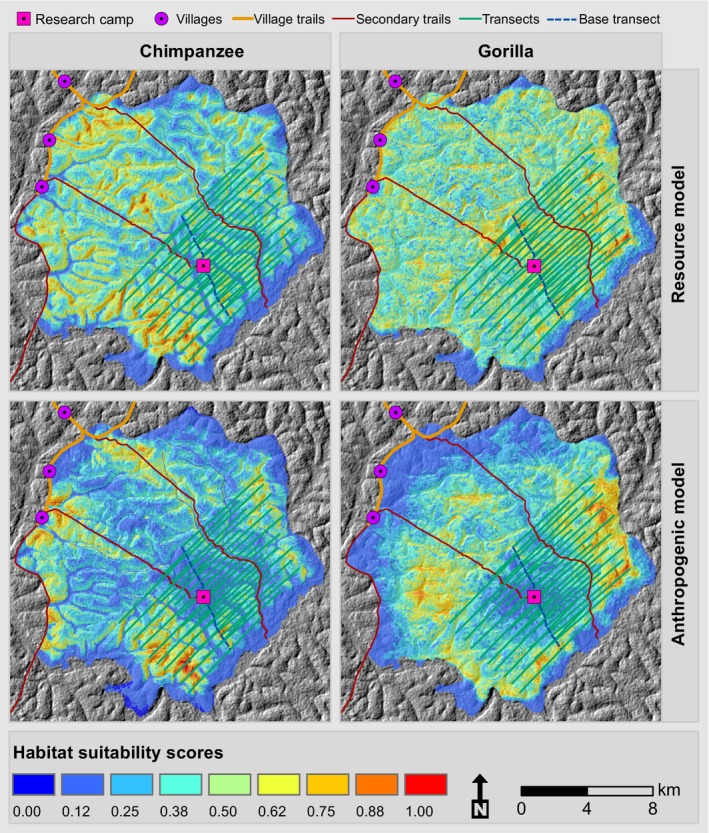
Great ape habitat suitability models displaying the spatial variation of suitability scores for gorillas and chimpanzees in two different models (resource and anthropogenic) throughout the landscape. The habitat suitability scores vary from 0 (low suitability) to 1 (high suitability). The background of each panel is a hillshade of the digital elevation model of the area. Each map contains the location of each variable considered in the analyses (villages, research, camp and trails) and the transects used for research

It is worth noting that the inclusion of human‐related variables in the models improved the predictions by increasing the values of AUC from 0.688 to 0.773 for chimpanzees and from 0.548 to 0.676 for gorillas (Figure 3). Habitat suitability maps show that without human presence, the study area was more suitable for gorillas than for chimpanzees and that shallows and rivers were not suitable for chimpanzee nesting; however, chimpanzees nested very close to rivers (Figure 5). In the resource model, the distribution of high suitability scores, while being heterogeneous, covered almost the entire study area for both gorillas and chimpanzees. However, the habitat suitability maps in the anthropogenic model show that for both gorillas and chimpanzees, some areas that were suitable in the fundamental niche model were no longer suitable in the anthropogenic model (Figure 5). For both gorillas and chimpanzees, the area at the center of the research site was not suitable when including anthropogenic variables in the models (Figure 5). Proximity to villages had more influence on gorillas than on chimpanzees, resulting in large areas unsuitable for gorilla nesting but not for chimpanzee nesting (Figure 5).

**Figure 6:**
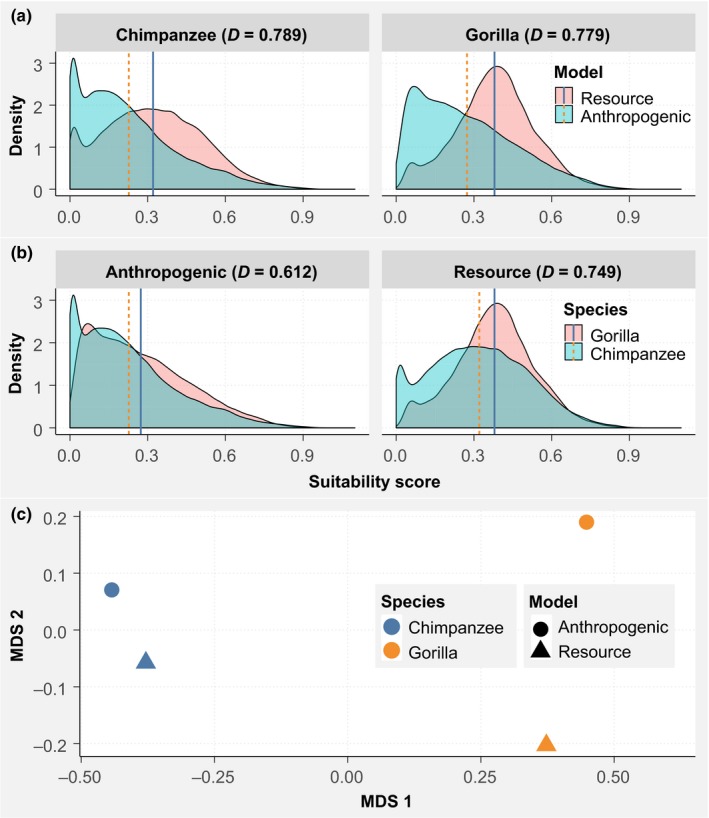
Overlap between the distributions of great apes in the resource and anthropogenic models. D = Schoener's index of overlap. (a) The overlap between the distributions from resource and anthropogenic models for chimpanzees and the overlap between the distributions from resource and anthropogenic models for gorillas and (b) the overlap between the distributions from the resource models of both gorillas and chimpanzees and overlap between distributions from the anthropogenic models of both gorillas and chimpanzees. The values obtained from the overlap analyses may be different from those observed in the graph because the index of overlap takes into account the spatial locations of suitability scores, while the graph only presents the density of those suitability scores. Each vertical line represents the average suitability score for the corresponding model. (c) Multidimensional scaling analysis (MDS) depicting the similarity between the different Maxent logistic outputs. Points that are closer to each other represent more similar distributions

The effects of anthropogenic variables on great ape distribution are highlighted in Figure 6. In the resource model, the distribution of chimpanzees included many pixels with suitability scores lower than 0.1, while the distribution of gorilla suitability scores followed a bell shape (Figure 6). The number of pixels where the suitability score decreased because of the inclusion of anthropogenic variables in the models was greater for gorillas than for chimpanzees; this is indicated by a lower overlap between distributions of gorillas and chimpanzees in both the resource and anthropogenic models (Figure 6a). The mean suitability score was lower for chimpanzees than for gorillas in both the resource and the anthropogenic models, and the difference between the mean suitability scores of gorillas and chimpanzees was higher in the resource models than in the anthropogenic models (Figure 6b); likewise, the suitability scores of the resource and anthropogenic models for chimpanzees were more similar than were the suitability scores of the resource and anthropogenic models for gorillas (Figure 6c). The overlap between distributions in the resource models of both gorillas and chimpanzees was greater than that of their distributions in the anthropogenic models (Figure 6c), suggesting that the distribution of gorillas was more affected by anthropogenic variables than was the distribution of chimpanzees.

## 4 DISCUSSION

Human presence and activities are changing the distribution of wildlife across many landscapes, indirectly by rendering parts of their habitat unsuitable as well as by directly removing individuals. Hence, to inform conservation measures that aim for species’ persistence in human‐dominated landscapes, human‐related variables need to be considered when predicting habitat suitability for wild animals. In this study, we evaluated the effects of forest use by people and the proximity of human settlements on great ape distribution by comparing simulated distributions in a system free from human disturbance (resource model) and a system with a risk of predation represented by human presence (anthropogenic model), and we determined how two sympatric species respond to that predation risk. The results indicate that in the resource model, habitat variables were important predictors of great ape nesting, while in the anthropogenic model, anthropogenic variables were the most important predictors of gorilla nesting, although habitat variables and elevation continued to be the most important predictors of chimpanzee nesting (Figure 3a,b). Human settlements and forest use reduced the suitability scores for gorilla habitats more than they did for chimpanzee habitats.

The major limitation of this study is the assumption of no phenological variation in fruit availability occurring. Fruit phenology changes seasonally and annually (Yamagiwa, Basabose, Kaleme, & Yumoto, 2008), and this may cause a seasonal change in the patterns of habitat selection and space use due to varying abundance of different fruiting species across habitats (Tédonzong et al., 2019). Also, the nest decay rate may also change with seasons (Binczik, Roig‐Boixeda, Heymann, & Waltert, 2019). However, we did not collect data on a seasonal basis to evaluate the effects of seasons (Tédonzong et al., 2019). Instead, we used a random sampling design, and our data collection spanned different seasons.

### 4.1 The effects of forest use and proximity to human settlements on great ape distribution

The most important predictors of chimpanzee habitat suitability in the resource model were elevation, chimpanzee‐preferred nesting habitat, distance to rivers, and slope; for gorillas, on the other hand, the most important predictors were gorilla‐preferred nesting habitat, surface relief ratio, chimpanzee‐preferred nesting habitat, and slope (Figure 4). Elevation was the highest contributor to the chimpanzee resource model, but this factor was not important in the gorilla resource model (Figure 3). The shape of the relationship between the probability of occurrence of chimpanzee nests and altitude was comparable to the bell (Figure 4) indicating that an increase in altitude of up to 675 m corresponded to an increase in the probability of chimpanzee nest occurrence, after which it started to decline. Fitzgerald, Coulson, Lawing, Matsuzawa, and Koops (2018) have found a similar shape for chimpanzees in the Greater Nimba Landscape, where the probability of occurrence started to decline at 900 m. At Gombe, the suitability of chimpanzee habitat followed the same shape irrespective of land cover classes with optimum values of suitability being found between 850 and 1,100 m of elevation (Foerster et al., 2016). In the Tofala Hill Wildlife Sanctuary, chimpanzee nests were found at high elevations (800–1,000 m) (Njukang, Angwafor, Richard, Akwanjoh, & Chuo, 2019). This indicates that elevation may be a good determinant for the choice of a nesting site by chimpanzees, where the areas with intermediate values of elevation are more suitable. In contrast, Etiendem, Funwi‐Gabga, Tagg, Hens, and Indah (2013) found that elevation was an important predictor of the Cross River gorilla distribution at Mawambi Hills.

These contrasting patterns do not allow us to generalize the importance of elevation as a predictor of nest building for either gorillas or chimpanzees. This corroborates the finding that elevation is a top predictor for many species but not all species (Hof, Jansson, & Nilsson, 2012). Nesting in high altitudes by chimpanzees in the Tofala Hill Wildlife Sanctuary (Cameroon) probably allowed them to minimize the encounter rates with logging and agricultural activities (Njukang et al., 2019). Apart from elevation in the chimpanzee model, the distribution and density of preferred nesting habitats were the most important predictors in the resource models for both gorillas and chimpanzees (Figure 3). The fact that chimpanzee‐preferred habitat was more important than gorilla‐preferred habitat in the resource and anthropogenic models for gorillas (Figure 3a,b) is an indication that gorillas interact with chimpanzee‐preferred habitat; gorillas only prefer nesting where the abundance of chimpanzee‐preferred habitat is low (Figure 4b).

Elevation and the normalized difference vegetation index (NDVI) were found to be the top predictor variables of chimpanzee fruiting species at Gombe National Park, Tanzania (Foerster et al., 2016). In the same park, NDVI was positively correlated with the time chimpanzees spent feeding (Pintea, 2007). Similarly, macaques (in Japan) mostly consumed fruits when they lived in a forest with higher NDVI (Tsuji, Ito, Wada, & Watanabe, 2015). We did not test for the contribution of fruiting trees in the model, but it is already known that chimpanzee‐preferred fruiting species are more abundant in chimpanzee‐preferred nesting habitats in the study site (Tédonzong et al., 2019). NDVI is generally used as a surrogate of vegetation greenness or plant productivity (Evans, James, & Gaston, 2006; McCormack, Zellmer, & Knowles, 2010; Williams, Bartholomew, Amakobe, & Githiru, 2018). Our method based on the determination of the density of habitat points represents a good alternative to NDVI. Analyses based on our method showed that areas with high densities of preferred nesting habitat points also correspond to areas with high densities of important fruiting plants for both gorillas and chimpanzees (Tédonzong et al., 2018). Additionally, the preferred plant species for both gorillas and chimpanzees are more abundant in chimpanzee‐preferred nesting habitats (Tédonzong et al., 2019).

Our findings indicate that anthropogenic variables (forest use and proximity to human settlements) were more important than habitat variables for gorillas but not for chimpanzees (Figure 3). Anthropogenic variables also improved the predictive power of the models, as shown by higher values of AUC in the anthropogenic models than in the resource models (Figure 3). These variables contributed to the decrease in the suitability of great apes' habitats (Figures 5 and 6). The reduction in the habitat suitability scores due to forest use and proximity to human settlements indicates that great apes may avoid areas with signs of human disturbance up to a certain level. Great apes may perceive a risk of predation when moving to areas disturbed by humans. The increase in AUC values in the anthropogenic models is an indication that human disturbance has modified the natural system and that the inclusion of human‐related variables is crucial to the evaluation of species' responses to their environment (Lindshield et al., 2017). This observation was made by several authors in previous studies (Alberti et al., 2003; Frid & Dill, 2002), and humans have been considered niche constructors in animal ecology (Albuquerque et al. (2018). Several authors have highlighted the relevance of human‐based variables in the prediction of wildlife habitats (Blom, Zalinge, Heitkönig, & Prins, 2005; Bowman, Ray, Magoun, Johnson, & Dawson, 2010; Koerner et al., 2017; Schuette, Wagner, Wagner, & Creel, 2013). We can liken the negative effects of humans on wildlife distribution to those of natural predators. Species may use the presence or absence of predators' odors as the main cue of the risk of predation (Salandre, Beil, Loehr, & Sundell, 2017). Great apes may use such cues to assess threats from humans in contexts where human activities, such as hunting, pose a direct threat (Setsaas, Hunninck, Jackson, May, & Røskaft, 2018; Storch, 2013). Also, the noise made by humans can contribute to frightening animals (Clinchy et al., 2016; Slabbekoorn, McGee, & Walsh, 2018).

The avoidance of human settlements or presence is often attributed to hunting; under such circumstances, animal species may find refuge where hunting intensity is low (Blom et al., 2005; Vanthomme et al., 2013). Although we did not measure hunting pressure in our analyses, the distance to human settlements was considered as a proxy for hunting pressure because the increasing distance to villages is likely to correspond to a decrease in hunting signs (Beirne et al., 2019; Koerner et al., 2017; Lhoest et al., 2019). Previous studies in our research site have shown that hunting signs, and not research activities, determined space use by gorillas and chimpanzees for nesting along transects (Tagg et al., 2018; Tagg & Willie, 2013). Within the research site, gunshots are frequently heard (authors’ observation); both ape species would likely respond negatively to such disturbance. Gorillas negatively responded to human forest use in the research site more than did chimpanzees; the difference between gorillas and chimpanzees may be due to different behavioral adaptations between the two animal species. A study of Ávila et al. (2019) revealed that our focal species, gorillas and chimpanzees, were not the target of hunters in our study area but that there has been a shift in hunting methods from traps to firearms between 2003 and 2016. It is, therefore, possible that hunting activities occurring in the research site may contribute to great ape distribution changes. In addition, a longitudinal study on the trend of wildlife populations around the villages of our study area showed that abundances significantly declined between 2002 and 2009 (Tagg et al., 2019), perhaps leading hunters to enter deeper into the forest (including in the research site) to hunt. This may explain why both gorillas and chimpanzees responded more to forest use in the research site than to the other human settlements (Figure 4c,d).

### 4.2 Influence of animal behavioral traits on their responses to human disturbance

Proximity to human settlements and human disturbance influenced chimpanzees relatively less than gorillas (Figures 4 and 6), indicating that chimpanzees may experience a trade‐off between finding fruits and avoiding perceived predation risk. This trade‐off has been documented in wildebeests (M'Soka et al., 2017) and red squirrels (Turkia, Korpimaki, Villers, & Selonen, 2018). If a species experiences a trade‐off between access to a resource and predation risk, it may avoid predation or areas of high predation risk when it has an alternative area in which to find food (Frid & Dill, 2002). This might explain why chimpanzees did not systematically avoid areas with human settlements as did gorillas (Figure 5). Although we collected data monthly, it is not possible to attest whether the use of areas near villages by chimpanzees was due to the absence of fruits in other areas. However, several studies have observed that chimpanzees move to villages to consume agricultural fruits when wild fruit availability is low in order to maintain their fruit‐dominated diet (Bryson‐Morrison, Matsuzawa, & Humle, 2016; McLennan, 2013). Those fruits were found to be relatively nutritious and procured energetic advantages to chimpanzees (McLennan & Ganzhorn, 2017). This behavioral flexibility has also been observed in many other primate species, such as *Colobus angolensis palliates* (Dunham, 2017) and *Cercopithecus albogularis labiatus* (Nowak, Wimberger, Richards, Hill, & Roux, 2017; Wimberger, Nowak, & Hill, 2017). For these species, the risk of starvation and sensitivity to fruit availability may be more important than the risk of predation induced by human disturbance (McLennan, 2013; Nowak et al., 2017; Weterings, Moonen, Prins, Wieren, & Langevelde, 2018). This implies that the nonavoidance of human settlements by chimpanzees may be a consequence of their pursuit of agricultural foods. In the present study site, however, agricultural foods have not yet been identified in the great ape diet (Petre, 2016; Tédonzong et al., 2018). Consequently, the consumption of agricultural foods may not be the reason for the nonavoidance of human settlements by chimpanzees. In addition, in our site, the villages are located where the densities of chimpanzee‐preferred nesting habitats and important fruits were high (Tédonzong et al., 2018), as depicted by the moderate correlation between the two variables (Figure S1). Hence, it can be concluded that in contrast to gorillas, chimpanzees are not highly negatively influenced by human settlements because of the presence of their preferred nesting habitats and wild fruits near villages.

In accordance with the present results, anthropogenic landmarks at Fongoli in Senegal did not prevent chimpanzees from visit fruiting trees, which were highly abundant in close proximity to human features (Lindshield et al. (2017). Similarly, human activities at Bili‐Uele (in the northern Democratic Republic of the Congo) had little effect on chimpanzees (Hicks, Roessingh, & Menken, 2012). At Bossou (Guinea), the high nutritional quality of agricultural fruits compensated for the stress induced by human presence in chimpanzees and reduced their concentration of glucocorticoid metabolite (McLennan, Howell, Bardi, & Heistermann, 2019). The fact that preferred nesting habitats and fruit species are more abundant near villages may reduce stress due to human presence in chimpanzees. However, the availability of forest fruits is subject to phenological changes. It was observed that, because chimpanzees at Bossou (Guinea) consumed crop foods when fruit availability is low, they did not split into smaller groups (Hockings, Anderson, and Matsuzawa (2012) as observed in other sites (Itoh & Nishida, 2007). Thus far, gorillas are known to form relatively stable groups (Watts, 2012). Reducing party size may be a strategy for chimpanzees to cope with periods of low fruit availability not compensated by crop foods, like at Bossou (Hockings et al., 2012). This is because chimpanzees are highly territorial (Herbinger, Boesch, & Rothe, 2001), and intraspecific competition is also high among them (Mitani, Watts, & Amsler, 2010). As a result, fatal attacks were observed between chimpanzees when population density was high (Mitani et al., 2010; Wilson et al., 2014).

Body size is a key aspect in predator–prey interactions; large‐sized animals may have a higher perception of the risk of predation than smaller‐sized ones (Davidson et al., 2009; McGraw & Zuberbuhler, 2008; Preisser & Orrock, 2012; Zuberbühler, 2007). Evidence of the effects of body size on the response of mammal species to human disturbance was recently reported in south‐east Cameroon (Lhoest et al., 2019) and Gabon (Beirne et al., 2019), where there was a gradient of increasing body mass of mammals with increasing distance to villages. The effects of body size on the response of different species to predation risk is related to the fact that different species present different flight initiation distances, and the flight initiation distance increases as body size increases (Gotanda, Turgeon, & Kramer, 2009; Møller & Erritzøe, 2014). Hence, the lower sensitivity of chimpanzees to human settlements than that of gorillas may be due to their smaller size. In an evaluation of the effects of noise caused by oil prospection on mammals in the Loango National Park, mammals with large home ranges (e.g. elephants) were found to be the most affected (Rabanal, Kuehl, Mundry, Robbins, & Boesch, 2010).

Chimpanzees are known to change their party size in response to fruit availability; they also increase in number to prevent gorillas from accessing fruiting trees (Basabose & Yamagiwa, 2002; Lehmann & Boesch, 2004). In Ugalla (western Tanzania), chimpanzees increased their party sizes in the evening to reduce the risk of predation by large nocturnal carnivores such as leopards (Ogawa, Idani, Moore, Pintea, & Hernandez‐Aguilar, 2007). The fission–fusion behavior of chimpanzees may be an advantage that allows them to cope with the presence of humans. Accordingly, evolutionary adaptations to competitive interactions may help species adapt to environmental change (Osmond & de Mazancourt, 2013).

Furthermore, chimpanzees are more arboreal than gorillas because they tend to build nests in trees, while gorillas tend to build nests on the ground; chimpanzees’ building of night nests at higher heights may represent an antipredator strategy (Stewart & Pruetz, 2013). For instance, in two adjacent sites with varying levels of predation risk, the height of chimpanzee nests was lower in the site with a lower level of predation risk than in the site with a higher level of predation risk (Pruetz et al., 2008). Additionally, chimpanzees in the Lebialem‐Mone Forest Landscape (Southwest Region, Cameroon) tend to build nests in trees in sites with high human population density and to build terrestrial nests in sites with low human population density (Last & Muh, 2013). Building arboreal nests in proximity to humans may constitute an advantage for chimpanzees in the form of reduced risk of predation. In Bili‐Uele (the northern Democratic Republic of the Congo), chimpanzees in trees withstood the presence of humans for a longer period than when they were terrestrial (Hicks et al., 2012). This is consistent with a study showing that the risk of predation may be higher for less arboreal than for more arboreal species (Wereszczuk & Zalewski, 2015). The differential responses to human settlements and forest use in gorillas and chimpanzees imply that the abundance of their populations may vary differently across space and time.

## 5 CONCLUSION

This study set out to evaluate the influence of human disturbance on great ape distribution and the implications of behavioral adaptations on their response to human disturbance. One of the more significant findings to emerge from this study is that the spatial distribution of gorillas was more negatively affected by distance to human settlements and forest use than was the distribution of chimpanzees. It was also shown that several behavioral and ecological adaptations (body size, intergroup interaction, terrestrial/arboreal, level of specialization/generalization, and competitive inferiority/superiority) may cause differing responses to human disturbance in gorillas and chimpanzees. The larger‐bodied species (gorilla) may perceive human presence as a greater risk, and the species with a high tolerance of intergroup encounters (gorilla) may find refuge in areas not disturbed by human presence without increasing intraspecific competition. The more generalist species (gorilla) may be more flexible if dispersed in areas where food availability is low, while the more specialized species (chimpanzee) may continue to use their preferred habitats despite the presence of human activities. The competitively dominant species (chimpanzee) exhibits a grouping pattern characterized by an increase in the number of individuals to defend food resources against competitors (gorilla); this strategy is an adaptive behavior that may help them respond to human presence. These findings enhance our understanding of the response of great ape species to the pressures experienced in a human‐dominated landscape and contribute to elucidating why different animal species respond differently to human disturbance. There is a link between the spatial distribution of animal species and their persistence (Oliver et al., 2012). As wild animals are increasingly exposed to human disturbance, understanding the resulting consequences of shifting species distributions and the long‐term survival of populations due to human disturbance will be of growing conservation importance.

## CONFLICT OF INTEREST

We have no conflict of interest to declare.

## AUTHOR CONTRIBUTION

LRDT, JW, and STM conceived the ideas and designed the methodology. LRDT collected the data, analyzed the data, and led the writing of the manuscript. All authors contributed critically to the drafts, contributed to, and approved the final manuscript.

### Open Research Badges

This article has earned an Open Data Badge for making publicly available the digitally‐shareable data necessary to reproduce the reported results. The data is available at https://doi.org/10.5061/dryad.f4qrfj6sk.

## Supporting information

Fig S1

## Data Availability

The presence data, the ASCII files of all the environmental data (and their respective projections), as well as the Maxent results (ASCII files and their respective projections), can be downloaded on Dryad, https://doi.org/10.5061/dryad.f4qrfj6sk (Tédonzong, Willie, Makengveu, Lens, & Tagg, 2020).
