# Supplementary figures and images for: Variation in behavioral traits of two frugivorous mammals may lead to differential responses to human disturbance
Source: Ecol Evol. 2020 Mar 11;10(8):3798–813. doi: 10.1002/ece3.6178 (PMC7160177; doi:10.1002/ece3.6178)

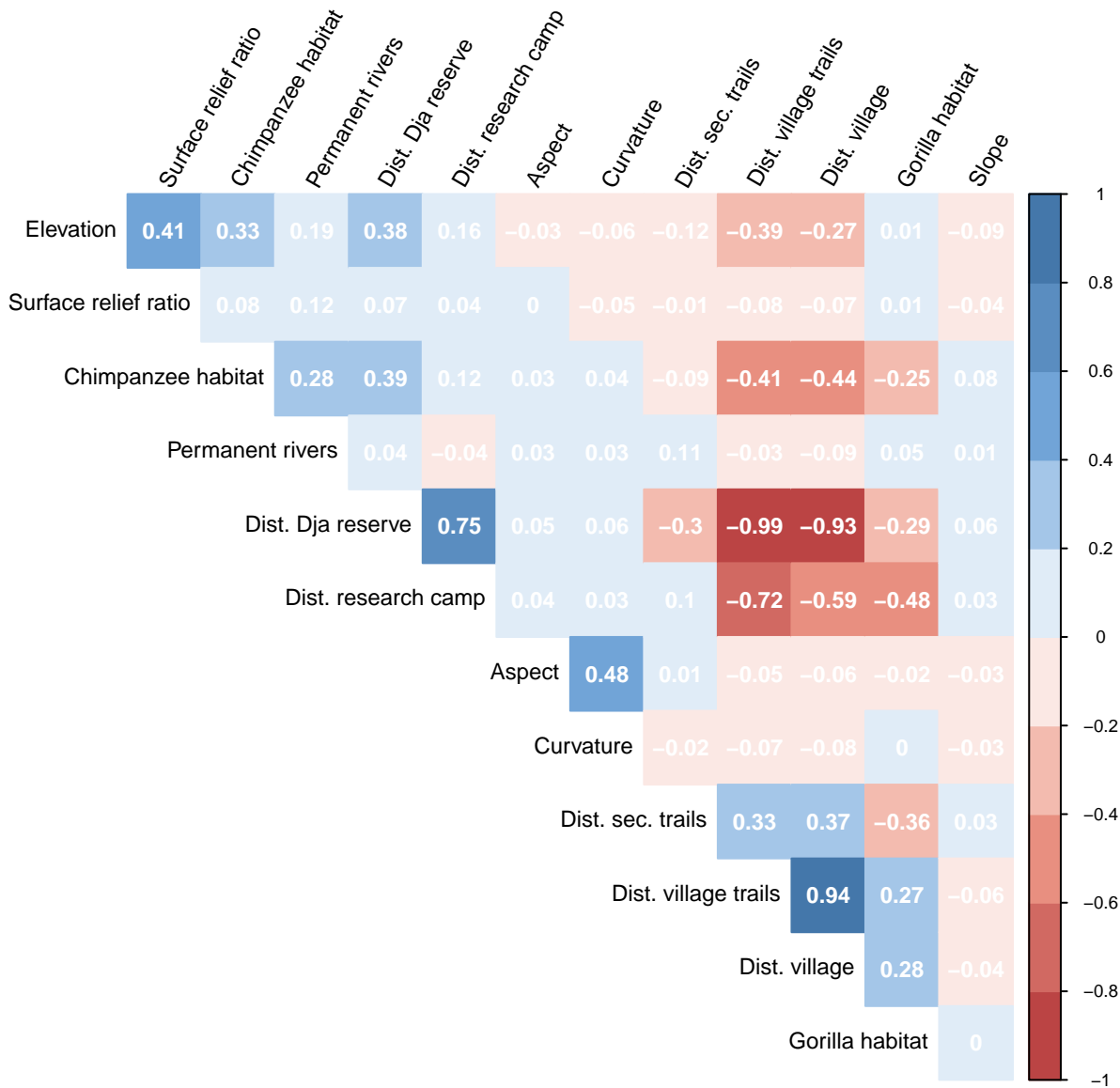

Supplement: Supplementary file 1 — Fig S1 [file ECE3-10-3798-s001.pdf]
